# Impact of cortactin in cancer progression on Wnt5a/ROR1 signaling pathway

**DOI:** 10.18632/oncotarget.28386

**Published:** 2023-03-21

**Authors:** Kamrul Hasan, Thomas J. Kipps

**Keywords:** cortactin, Wnt5a, ROR1, migration, metastasis

Cortactin is an intracellular cytoskeletal protein that can undergo tyrosine phosphorylation upon external stimulation and promote polymerization and the assembly of the actin filament that is required for cell migration [1]. Upon stimulation, cortactin binds and activates actin related protein Arp2/3 complex, a *de novo* actin nucleator that can induce F (filamentous)-actin polymerization [1, 2]. Cortactin (also known as EMS1 or CTTN) is expressed broadly in a variety of cancers, for which it plays an apparent role in cellular protrusions, which include lamellipodia and filopodia formation to promote migration and metastasis. Moreover, cortactin is expressed in (i) primary chronic lymphocytic leukemia (CLL) and primary breast-cancer cells, (ii) at least 15% of metastatic breast carcinomas, and (iii) CLL or breast-cancer cell-lines [3, 4]. In structure, cortactin contains a SH3 domain that allows it to bind characteristic motifs (-P-X-X-P-), which can be found in the proline-rich-domains (PRD) of other proteins, including ROR1 [1, 3, 4].

The receptor-tyrosine-kinase-like orphan receptor 1 (ROR1) is a developmentally-restricted, evolutionarily conserved, type-I membrane protein, which comprises (1) an extracellular domain with a frizzled-like, cysteine-rich subdomain and (2) a cytoplasmic domain, comprising a tyrosine-kinase-like subdomain, two serine/threonine-rich subdomains, and a proline-rich subdomain [3]. Expression of ROR1 attenuates during fetal development and is negligible on most normal adult tissues [5]. We have observed that ROR1 can serve as a receptor for Wnt5a that could induce non-canonical Wnt signaling [3, 6].

We have found (as have other investigators) that ROR1 is expressed by a variety of human cancers, which include CLL and breast cancer, suggesting that ROR1 may play a role in cancer pathogenesis [3–5]. Consistent with this notion, other studies have shown that expression of ROR1 can enhance disease progression, tumor-cell growth, epithelial-mesenchymal transition (EMT), migration, and metastasis in CLL or breast cancer [3, 4]. Moreover, high-level tumor-cell expression of ROR1 is associated with adverse outcomes among patients with CLL, breast cancer, or other cancers [6–10].

**Figure 1:**
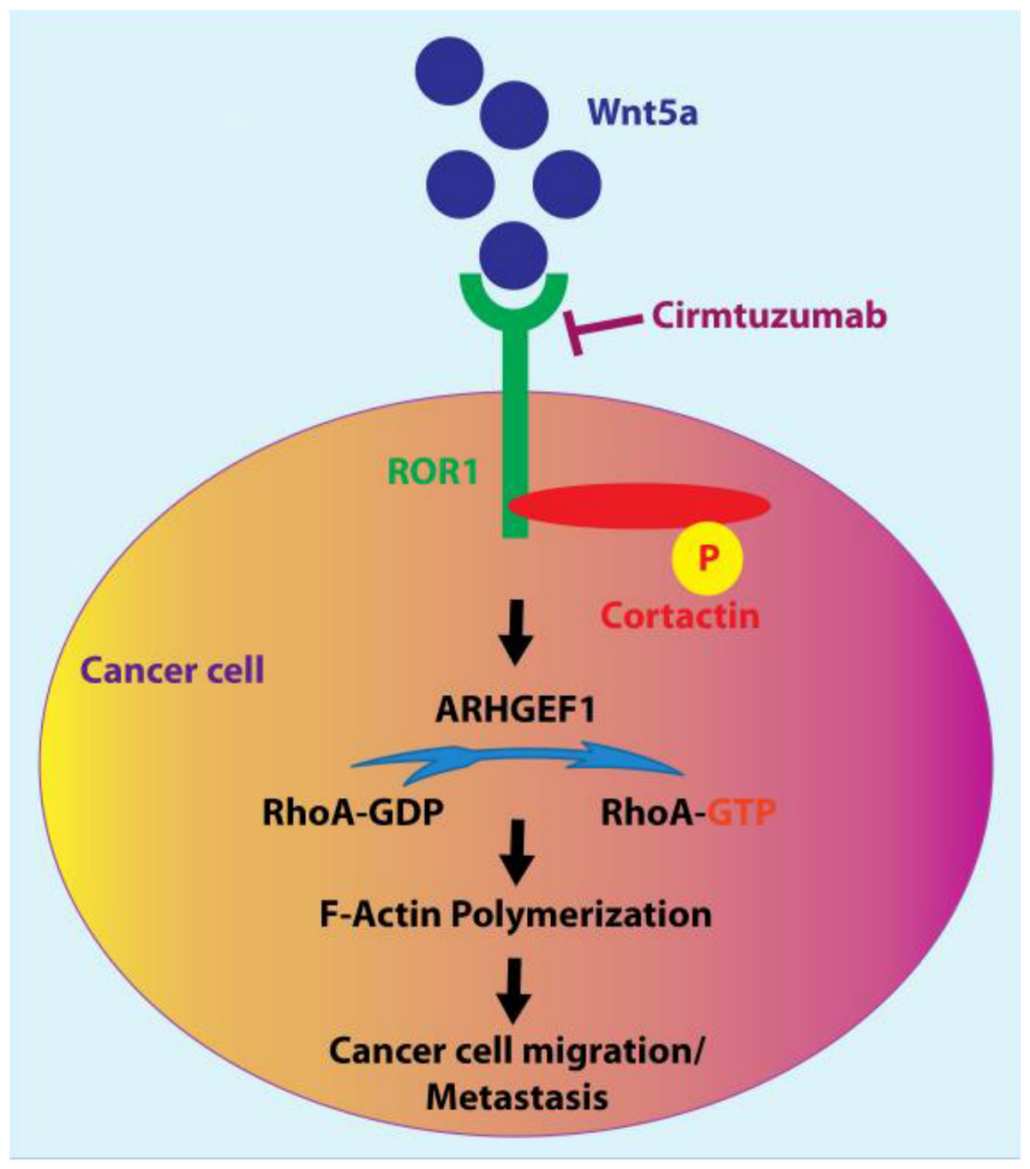
The molecular mechanism(s) of how Wnt5a signaling could (i) induce ROR1 and cortactin association, tyrosine phosphorylation of cortactin, and activation of RhoA, (ii) enhance F-actin polymerization, and (iii) subsequently promote migration or metastasis of cancer cells.

Recently, we described that cortactin, which is abundantly expressed in CLL B cells and activated at a higher level (*p* < 0.001) in ROR1^Pos^ (i.e., high-level expression of ROR1) compared to ROR1^Neg^ (i.e., low to negligible expression of ROR1) CLL [3]. Moreover, in freshly isolated CLL cells, we have found that cortactin associates with ROR1. This suggests that Wnt5a could induce ROR1 to complex with cortactin, which then undergoes tyrosine phosphorylation at Y421, recruits ARHGEF1, and activates RhoA, thereby enabling leukemia-cell migration (Figure 1). Indeed, although such effects could not be inhibited by ibrutinib (a BTK inhibitor), they could be blocked by zilovertamab (previously known as cirmtuzumab or UC-961), a humanized anti-ROR1 mAb, which is under phase-II clinical testing among patients with CLL (https://clinicaltrials.gov/ct2/show/NCT02222688). These findings have been corroborated in the CLL cell line MEC1 expressing ROR1.

We generated truncated forms of ROR1 and found its proline-rich domain (PRD) was necessary for this interaction. Accordingly, single amino-acid substitutions were introduced of proline (P) to alanine (A) in the ROR1-PRD at positions 784, 808, 826, 841, or 850 in potential SH3-binding motifs. In contrast to wild-type ROR1 or other ROR1^P≥A^ mutants, ROR1^P(841)A^ exhibited impaired capacity to recruit cortactin and ARHGEF1 to ROR1 in response to Wnt5a. Moreover, Wnt5a could neither induce cells expressing ROR1^P(841)A^ to phosphorylate cortactin nor enhance CLL-cell F-actin polymerization. Overall, these results reveal that cortactin plays an important role in Wnt5a-enhanced CLL cell migration/F-actin polymerization via a ROR1-dependent signaling pathway, which cannot be inhibited by ibrutinib or other inhibitors of BTK.

We also reported that using breast cancer patient-derived xenografts (PDX) or cell lines that Wnt5a induces ROR1 to recruit cortactin, which undergoes phosphorylation at Y421, not only recruits/activates ARHGEF1, but also activates RhoA to promote breast-cancer-cell migration/metastasis [4]. The humanized anti-ROR1 mAb zilovertamab could block such effects, which supports its evaluation in the treatment of patients with breast cancer (https://clinicaltrials.gov/ct2/show/NCT02776917). Against this backdrop, the phosphorylation/activation status of cortactin could be a surrogate marker to select dosage regimen and examine functional effects of zilovertamab in clinical trials of CLL or breast cancer patients. Moreover, these signaling mechanism(s) could be effective in other cancers that express ROR1. Furthermore, zilovertamab could be a new therapeutic development to block such effects [5–10].
